# Central Projection of Antennal Sensory Neurons in the Central Nervous System of the Mirid Bug *Apolygus lucorum* (Meyer-Dür)

**DOI:** 10.1371/journal.pone.0160161

**Published:** 2016-08-01

**Authors:** Gui-Ying Xie, Xin-Cheng Zhao, Bai-Wei Ma, Pei Guo, Guo-Ping Li, Hong-Qiang Feng, Guo-Liang Wu

**Affiliations:** 1 Department of Pesticide, College of Plant Protection, Henan Agricultural University, Zhengzhou, 450002, China; 2 Department of Entomology, College of Plant Protection, Henan Agricultural University, Zhengzhou, 450002, China; 3 Henan Key Laboratory of Crop Pest Control, Key Laboratory of Integrated Pest Management on Crops in Southern Region of North China, International Joint Research Laboratory for Crop Protection of Henan, Institute of Plant Protection, Henan Academy of Agricultural Sciences, Zhengzhou, 450002, China; 4 Department of Pomology, College of Horticulture, Henan Agricultural University, Zhengzhou, 450002, China; University of Arizona, UNITED STATES

## Abstract

The mirid bug *Apolygus lucorum* (Meyer-Dür), a polyphagous pest, is dependent on olfactory cues to locate various host plant species and mates. In this study, we traced the projection pathway of the antennal sensory neurons and visualized their projection patterns in the central nervous system of *A*. *lucorum* through confocal microscopy and digital reconstructions. We also examined the glomerular organization of the primary olfactory center of the brain, the antennal lobe, and created a three-dimensional model of the glomeruli. We found that the axons of the sensory neurons project into the brain via the ipsilateral antennal nerve, and descend further into the gnathal ganglion, prothoracic ganglion, mesothoracic ganglion, and metathoracic ganglion, and reach as far as to the abdominal ganglion. Such a projection pattern indicates that antennal sensory neurons of *A*. *lucorum* may be potentially directly connected to motor neurons. The antennal lobe, however, is the major target area of antennal sensory neurons. The antennal lobe is composed of a large number of glomeruli, i.e. 70–80 glomeruli in one AL of *A*. *lucorum*. The results of this study which provide information about the basic anatomical arrangement of the brain olfactory center of *A*. *lucorum*, are important for further investigations of chemosensory encoding mechanisms of the mirid bug.

## Introduction

The mirid bug *Apolygus lucorum* (Meyer-Dür.) (Heteroptera:Miridae) is a polyphagous pest, feeding on about 200 different plant species, including cotton, cereals, vegetables, and fruit trees [1, 2]. Outbreak of *A*. *lucorum* occurred in China during the previous decade, which was facilitated by the long-term wide-scale adoption of transgenic *Bacillus thuringiensis* cotton [3]. In order to develop eco-friendly and effective strategies to control this pest, a great attention was paid by many scientific researchers to explore the knowledge of biology of *A*. *lucorum* in a variety of aspects, such as life history, development, diapause, flight and dispersal capacity, mating, oviposition, as well as host plant selection [4–6].

As in other insect species, it has been demonstrated that olfaction plays an essential role for *A*. *lucorum*, both in selecting host plants for food and finding mates for reproduction [7–10]. Adults of *A*. *lucorum* prefer host plants at the flowering stage and can effectively locate the flowering plants that bloom spatially and temporally differently [9]. Such alternations among host plants at the flowering stage are based on detection of distinct volatile compounds, such as m-xylene, butyl acrylate, butyl propionate, and butyl butyrate. Furthermore, female-released sex pheromones mediate mate location of male *A*. *lucorum*. Four compounds, hexyl butyrate, (E)-2-hexenyl butyrate, (E)-4-oxo-2-hexenal, and (E)-2-octenyl butyrate have been identified as female sex pheromones of *A*. *lucorum* [8, 10]. The mixture of (E)-2-hexenyl butyrate and (E)-4-oxo-2-hexenal is shown to attract males of *A*. *lucorum* in China [8], while four compounds are essential for attraction of the Korean population [10]. In addition, it has been demonstrated that the minor component (E)-2-octenyl butyrate, can increase attraction of *A*. *lucorum*, and inhibit attraction of other mirid bugs, *A*. *spinolae*, *Orthops campestris*, and *Stenotus rubrovittatus*, thus serving as an interspecific signal for the isolation of sympatric species [10].

The relevant odour cues are first detected at the periphery by olfactory sensory neurons being housed in antenna sensilla. Results from electroantennographical tests have indicated that the antennal of *A*. *lucorum* can detect volatiles of host plant and sex pheromone components [7, 9]. At the molecular level, odorant binding proteins (OBPs), and odorant receptors (ORs) detecting the key odour cues have been identified and characterized in *A*. *lucorum* [11–13]. The peripheral signals are then conveyed to the central nervous system (CNS) to ultimately induce behavioral responses. To understand how olfactory information is processed in the nervous system of *A*. *lucorum* it is critical to elucidate olfaction-based behaviors. However, the olfactory pathway of *A*. *lucorum*, from input to output, is largely unknown.

In this study, we traced the projection pathway of the antennal sensory neurons and visualized their innervation patterns in the CNS of *A*. *lucorum* through fluoresecent staining combined with confocal microscopy and digital reconstructions. We also examined the glomerular organization of the primary olfactory center of the brain, the antennal lobe (AL), and created a three-dimensional model of the AL. These results may provide new knowledge being important for further investigation of chemosensory encoding mechanisms in the brain of *A*. *lucorum*.

## Materials and Methods

### Insect rearing

*A*.*lucorum* adults (male and female), which were used to establish a colony in the laboratory, were originally collected from a cotton field at the Henan Research and Experiment Station for Modern Agriculture (35°0'13.24''N, 113°42'28.88''E) of Henan Academy of Agricultural Sciences, Yuanyang, Henan province, China. The laboratory colony of *A*. *lucorum* was reared in aerated plastic boxes (20cm×15cm×10 cm), fed on green bean pod (*Phaseolus vulgaris*) under the conditions of 28±1°C, 60% relative humidity, and a 16 h:8 h illumination regime. Seven to ten days old male adults, after eclosion, were used for the experiments. No permission is needed for the use of *A*. *lucorum* in experiment according to Chinese law of animal welfare.

### Anterograde fills of antennal nerves

In order to examine the projection pathway of sensory neurons from the antennal sensilla, backfill stainings were performed. The animals were immobilized in a plastic tube with the head exposed outside the tube. One antenna was cut at the base of the scapus and crystals of the fluorescent dye Micro-Ruby (tetramethylrhodamine dextran with biotin, Micro-Ruby, Molecular Probes; Invitrogen, Eugene, OR) were then placed by using a needle at the cut end. The cut surface was covered with Vaseline and the animal was placed in the dark in Petri dishes with a moist filter paper, at 4°C overnight, for allowing transportation of the dye in the sensory axons. After the animal was briefly cooled on ice, the brain and ventral nerve cord was dissected out in Ringer’s saline for synapsin immunostaining as described below.

### Immunocytochemistry

In order to visualize the central projection pathway of the antennal sensory neurons, anti-synapsin antibody staining of the neuropil structures was performed. The dissected brain and ventral nerve cord which contained pre-stained sensory axons was transferred into 4% paraformaldehyde in 0.1 M phosphate-buffered saline (PBS, pH 7.4) to be fixed overnight at 4°C. Following rinse in PBS (4×15minutes), preincubation was performed with 5% NGS (Sigma, St. Louis, MO) in 0.1M PBS containing 0.5% Triton X-100 (PBST; 0.1 M, pH 7.4) overnight at 4°C. The primary antibody SYNORF1 (Developmental Studies Hybridoma Bank, University of Iowa), at a concentration of 1:100 (with 5% NGS in PBST) was applied at 4°C for 5 days. Following six rinses in PBS (20 min, each), incubation in the secondary antibody, Cy2-conjugated anti-mouse (Invitrogen, Eugene, OR; dilution 1:300 with 1% NGS in PBST), was performed for 3 days at 4°C. Finally, the CNS was washed 6 × 20 min in PBS, dehydrated with an ascending ethanol series, cleared in methylsalicylate, and mounted in Permount in a perforated aluminium slide with two glass coverslips.

The anti-synapsin antibody staining was also performed on sections of the entire *A*.*lucorum* trunk to reveal the location and ganglion composition of the CNS. The animal was briefly cooled on ice, then transferred into 4% paraformaldehyde in 0.1M PBS overnight at 4°C. After the ablation of wings, legs and mouthparts the body was embedded in albumin-gelatin (12.4% ovalbumin and 4.6% gelatin) and postfixed in 4% formaldehyde solution overnight at 4°C. The animal was cut into sections at thicknesses of 100 μm by using a vibrating blade microtome (Leica vt1000S, Wetzlar, Germany). The sections were rinsed with 0.1M PBS (3×10 minutes), preincubated with 5% NGS for 30 min at room temperature, and incubated with SYNORF1 at a concentration of 1:100 for 3 d at 4°C. After washing in 0.1 M PBS (3×10 min), the incubation with the secondary antibody, Cy2-conjugated anti-mouse (1:300 with 1% NGS in PBST) was performed for 24 h at 4°C was performed. Then the sections were rinsed in 0.1M PBS (3×10 min), dehydrated in an increasing ethanol series (50%, 70%, 90%, 95%, 99%, 100% × 2, 3 min each), cleared in Xylene for 5 min, and mounted on the slide with Permount.

### Confocal image acquisition and three-dimensional reconstructions

All images were obtained with a confocal laser scanning microscope (LSM 780, META Zeiss, Jena, Germany) with objectives of 10 × (Plan-Neofluar 10×/0.3) and 20 × (Plan-Neofluar 20×/0.5l). A 488-nm line of an Argon laser was used to excite the Cy2 and a HeNe1 laser 543-nm line to excite the Micro-Ruby. The confocal images were obtained with the resolution of 1024×1024 voxels at intervals of 3 or 4 μm.

For three-dimensional reconstruction, each neuropil was labeled by using the Amira software (AMIRA 5.3, Visage Imaging, Fürth, Germany). The neuropil structures were labeled as previously described by using the segmentation editor, including the “brush” and “interpolate” tools [14, 15]. Also, three-dimensional reconstructions of all glomeruli in the antennal lobes were created and their individual volumes calculated.

### Terminology

The ganglia of the CNS were named according to previous reports of hemipteran insects [16–18]. The neuropil structures of the brain were named as suggested by Ito et al [19]. The orientation of the CNS refers to the axis of the insect body.

## Results

### The central nervous system of *A*. *lucorum*

**Fig 1:**
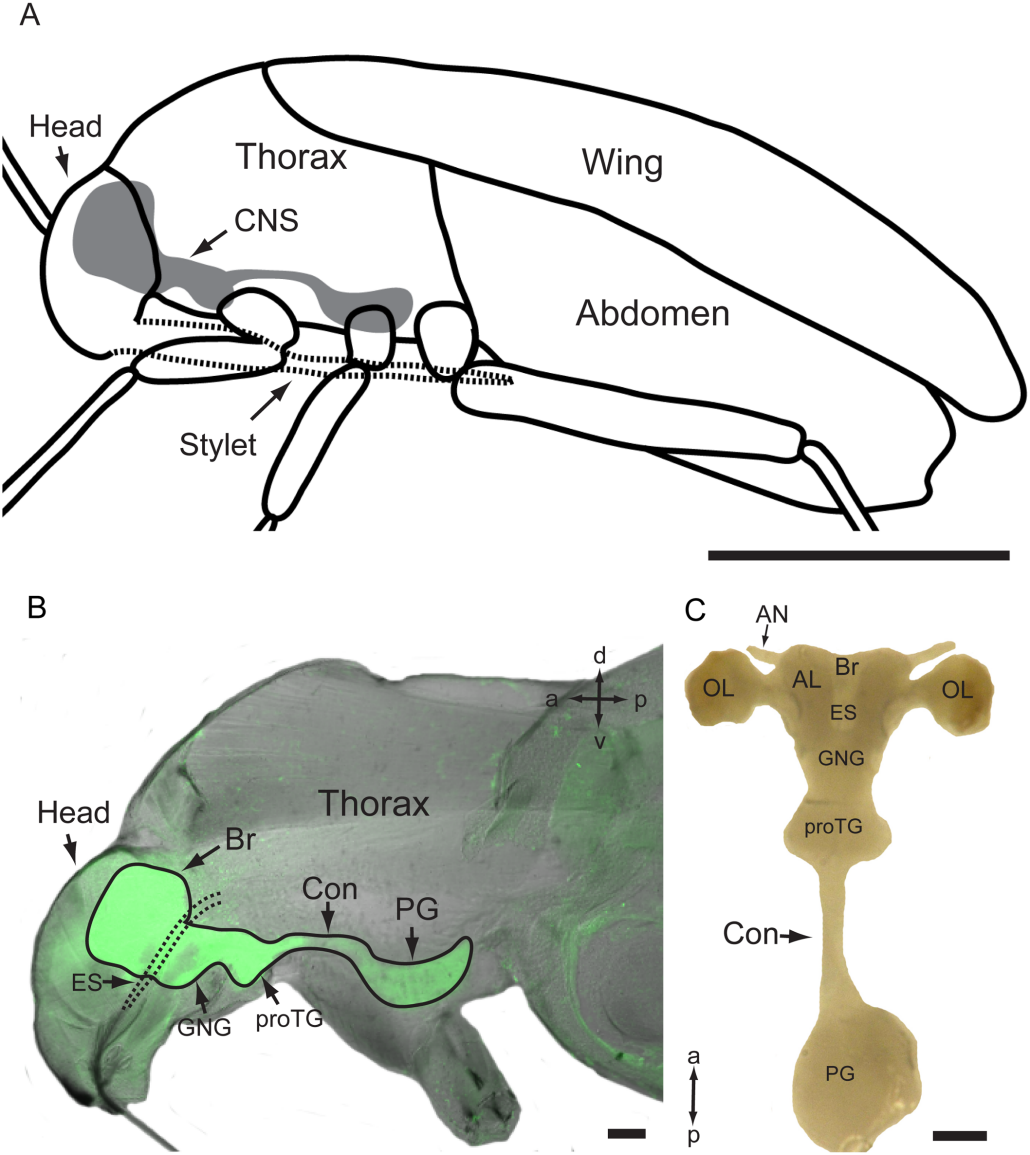
The central nervous system of *A*.*lucorum*. (A) Diagram of the body of *A*. *lucorum* showing the location and composition of the central nervous system (CNS). (B) Confocal image of the section showing the location and composition of the CNS in the body of *A*. *lucorum*. (C) Dissected CNS with all ganglia. AN, antennal nerve; AL, antennal lobe; Br, brain; CNS, central nervous system; Con, connective; ES, esophagus; GNG: gnathal ganglion; OL, optic lobe; PG, posterior ganglion; proTG, prothoracic ganglion. Directions: a, anterior; d, dorsal; l, lateral; p, posterior, v, ventral. Scale bars: 1 mm in A, 100 μm in B and C.

The CNS of *A*. *lucorum* is composed of the brain and the ventral nerve cord (Fig 1A and 1B). The brain is positioned above the esophagus, in the dorso-posterior part of the head capsule (Fig 1B). The ventral nerve cord which is situated under the esophagus, is formed by the gnathal ganglion (GNG), prothoracic ganglion (proTG), and posterior ganglion (PG) (Fig 1B). The GNG is located in the ventro-posterior part of the head capsule, the proTG near the prosternum, and the PG near the mesosternum (Fig 1B). The brain, GNG, and proTG are fused together, forming a brain-GNG-proTG complex, while the PG is set far away from the complex, linked by a single paired connective to the proTG (Fig 1B and 1C).

**Fig 2:**
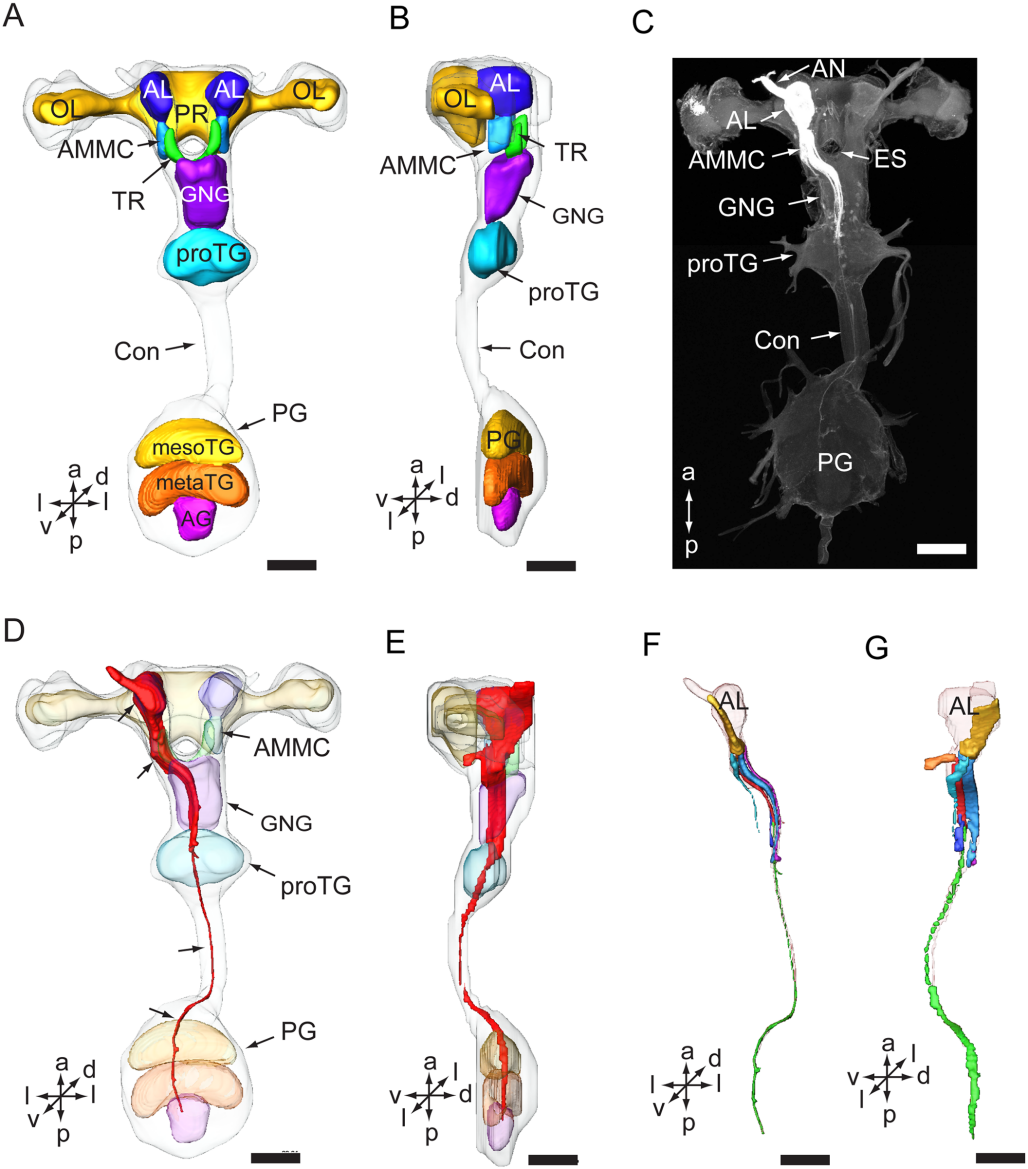
Three-dimensional reconstructions including antennal sensory pathways in the central nervous system. (A) Three-dimensional reconstruction model of the CNS in a ventral view. (B) Three-dimensional reconstruction model of the CNS in a lateral view. (C) Confocal image showing the antennal sensory pathway in the CNS. (D) Three-dimensional reconstruction model showing the antennal sensory pathway (indicated by arrows) in the CNS in a ventral view. (E) Three-dimensional reconstruction model showing the antennal pathway in the CNS in a lateral view. (F) Three-dimensional reconstruction of tracts of antennal sensory neurons in a ventral view. (G) Three-dimensional reconstruction of tracts of antennal sensory neurons in a lateral view. AG, abdominal ganglion; AN, antennal nerve; AL, antennal lobe; AMMC, antennal mechanosensory and motor center; Con, connective; ES, esophagus; GNG: gnathal ganglion; mesoTG, mesothoracic ganglion; metaTG, metathoracic ganglion; OL, optic lobe; PE, protocerebrum; PG, posterior ganglion; proTG, prothoracic ganglion; TR, tritocerebrum. Directions: a, anterior; d, dorsal; l, lateral; p, posterior, v, ventral. Scale bars: 100 μm.

The brain consists of the protocerebrum (PR), deutocerebrum (DE), and tritocerebrum (TR) [15]. The PR is located posteriorly in the brain (Fig 2A and 2B). The optic lobes (OL) form the lateral parts of the PR (Fig 2A and 2B). The DE is located ventrally of the PR and consists of the antennal lobe (AL) and the antennal mechanosensory and motor center (AMMC) (Fig 2A and 2B). The TR is located ventrally of AMMC, on either side of the esophagus foramen (Fig 2A and 2B). The GNG is located ventral-posteriorly of the TR and the esophagus, and consists of three fused neuromeres, the mandibular, maxillary, and labial neuromere [15]. The proTG is a single ganglion, located posteriorly of the GNG. The PG is a fused ganglion as well, consisting of the mesothoracic ganglion (mesoTG), metathoracic ganglion (metaTG), and abdominal ganglion (AG) (Fig 2A and 2B) [15].

### Central projections of the antennal sensory neurons

From 43 attempted trials, 14 preparations were successfully stained. The axons of the antennal sensory neurons project via the antennal nerve to the ipsilateral side of several neuropils in the CNS, including, from anterior to posterior, the AL, AMMC, GNG, proTG, mesoTG, metaTG, and AG (Fig 2C–2E). The staining was strongest in the AL and became weaker in the posterior regions.

**Fig 3:**
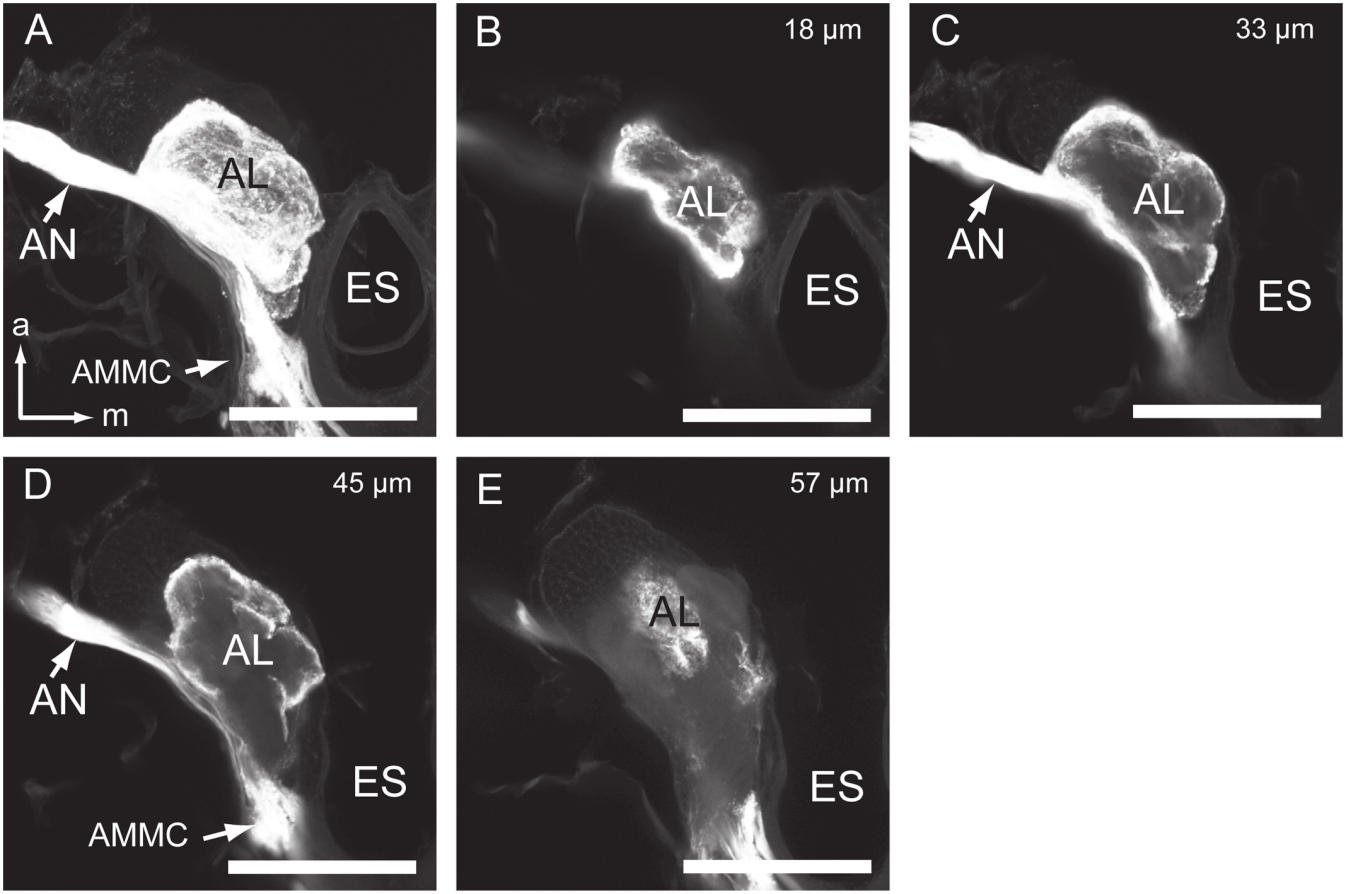
Confocal images of the antennal lobe with staining of antennal sensory neurons. (A) Confocal image of the AL showing the innervation of antennal sensory neurons throughtout the AL. (B-E) Confocal images of the AL with staining of antennal sensory neurons at different depths. (B) 18 μm. (C) 33 μm. (D) 45 μm. (E) 57 μm. AN, antennal nerve; AL, antennal lobe; AMMC, antennal mechanosensory and motor center; ES, esophagus. Directions: a, anterior; m, medial. Scale bars: 100 μm.

**Fig 4:**
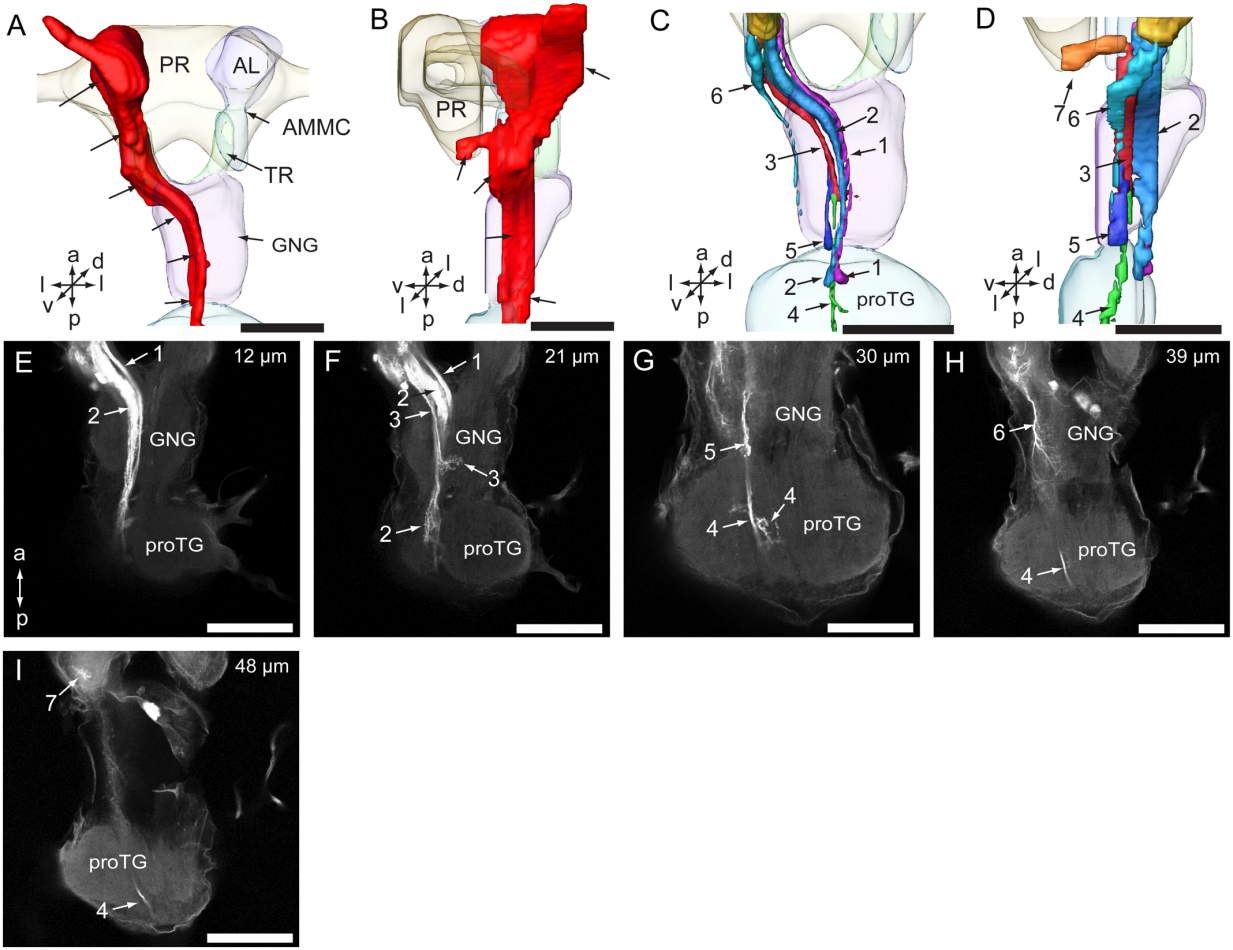
Close view of different projections of antennal sensory pathways in the brain, gnathal ganglion, and prothoracic ganglion. (A) Three-dimensional reconstruction model showing the antennal sensory pathway (indicated by arrows) in the brain, GNG, and proTG in a ventral view. (B) Three-dimensional reconstruction model showing antennal sensory pathways (indicated by arrows) in the brain, GNG, and proTG in a lateral view. (C) Three-dimensional reconstruction model showing different tracts of antennal sensory pathway in the brain, GNG, and proTG in a ventral view. (D) Three-dimensional reconstruction model showing different tracts of antennal sensory pathways in the brain, GNG, and proTG in a lateral view. (E-I) Confocal image of the GNG and proTG with different tracts of antennal sensory pathway at different depths. (E) 12 μm. (F) 21 μm. (G) 30 μm. (H) 39 μm. (I) 48 μm. GNG, gnathal ganglion; proTG, prothoracic ganglion. Directions: a, anterior; d, dorsal; l, lateral; p, posterior, v, ventral. Scale bars: 100 μm.

**Fig 5:**
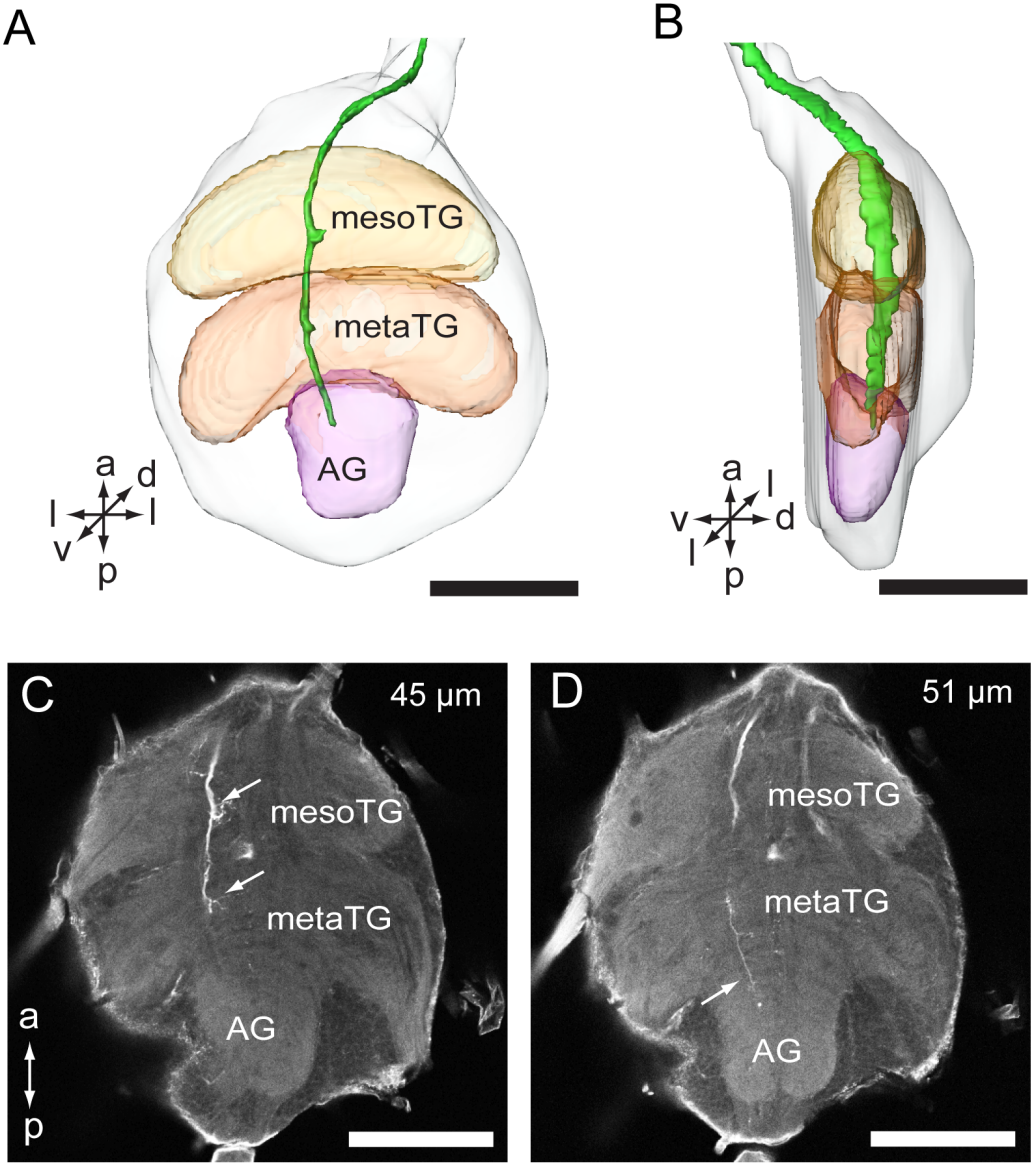
Close view of projections of antennal sensory pathway in posterior ganglion. (A) Three-dimensional reconstruction of the PG with axons of antennal sensory neurons (ventral view). (B) Three-dimensional reconstruction model of the posterior ganglion with axons of antennal sensory neurons (lateral view). (C) Confocal image of the PG at the depth of 45 μm showing axons of antennal sensory neurons (indicated by arrows) in the mesoTG and metaTG. (D) Confocal image of the PG at the depth of 51 μm showing axons of antennal sensory neurons (indicated by an arrow) in the AG. AG, abdominal ganglion; mesoTG, mesothoracic ganglion; metaTG, metathroacic ganglion. Directions: a, anterior; d, dorsal; l, lateral; p, posterior, v, ventral. Scale bars: 100 μm.

The antennal nerve is divided into two large bundles, one projecting into the AL, and the other projecting further down along the lateral side of the AL. In the AL, most glomeruli (if not all) receive projections from the antennal sensory neurons. The staining pattern is characterized by axon terminals targeting the cortex of the glomeruli (Fig 3). The remaining axon bundle is divided further into several tracts projecting into different neuropils, but not the tritocerebral area (Figs 2F, 2G, 4A and 4B). There are at least 7 tracts observed (Fig 4C–4I). Tracts 1 and 2 run medially of the GNG and terminate in the antero-ventral part of the proTG (Fig 4C–4F). Tract 2 is much thicker than Tract 1. Fine arborizations are given off at the terminal of both tracts. Tract 3 is positioned lateral to Tract 2 and targets the medial line of the GNG (Fig 4C–4F). Tract 4 runs along with Tract 3 and projects further to the proTG and PG (Figs 4C and 5). Tract 4 gives off fine arborizations in the proTG, mesoTG, metaTG, and AG (Figs 4G, 5C and 5D). Tract 5 runs along with Tract 3 as well and terminates in the area between the GNG and proTG (Fig 4C, 4D and 4G). Tract 6 targets mainly the AMMC, but a small sub-bundle projects to the latero-ventral part of the GNG (Fig 4C, 4D and 4H). Tract 7 is short and projects dorsally to the posterior slope of the protocerebrum (Fig 4D and 4I).

### Glomerular organization of the antennal lobe

**Fig 6:**
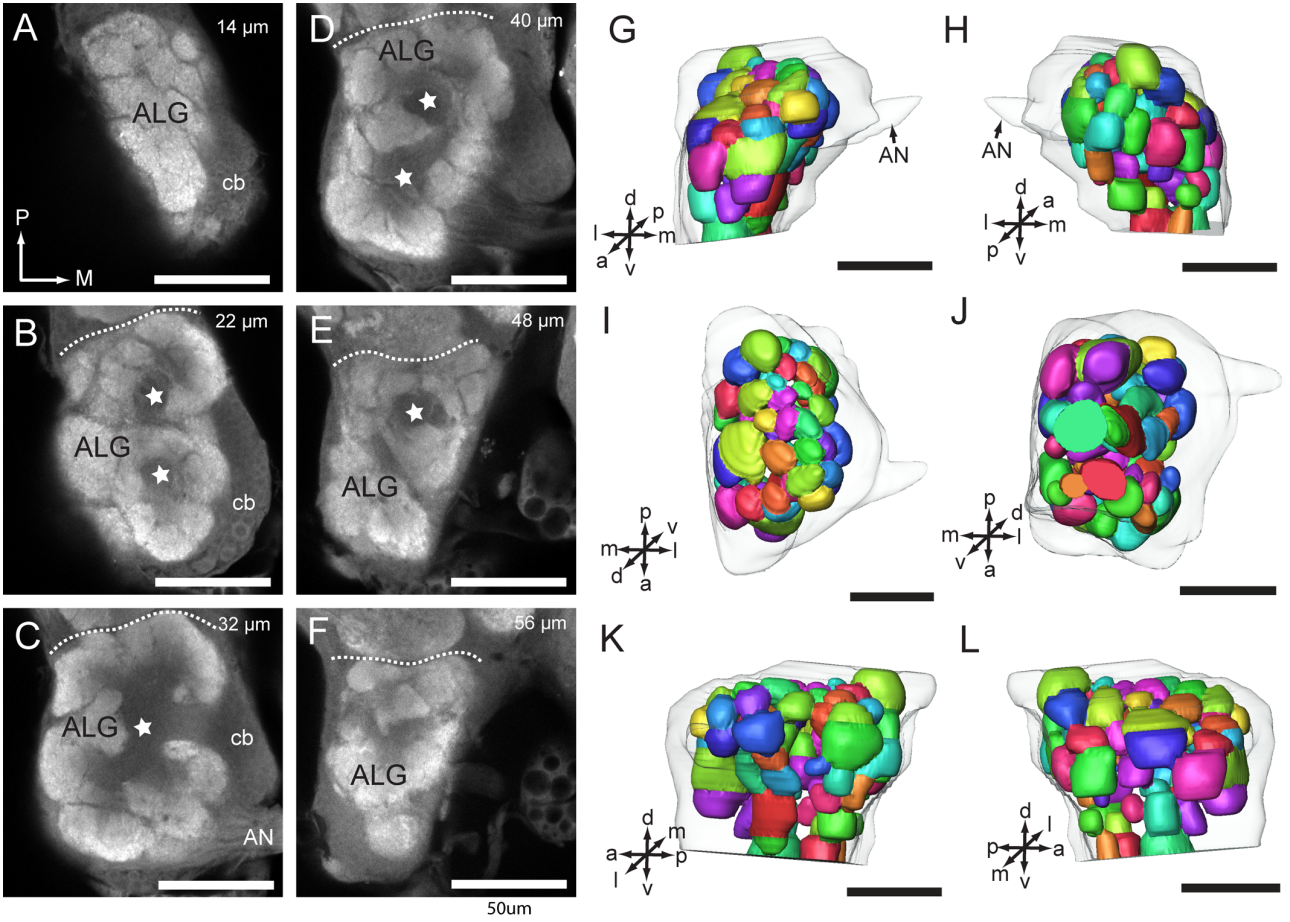
Confocal images and three-dimensional reconstructions of the antennal lobe glomeruli. (A-F) Confocal images of AL sections at different depths. (A) 14 μm. (B) 22 μm. (C) 32 μm. (D) 40 μm. (E) 48 μm. (F) 56 μm. (G-L) Three-dimensional reconstructions of the AL in different views. (G) anterior view. (H) posterior view. (I) dorsal view. (J) ventral view. (K) lateral view. (L) medial view. ALG, antennal lobe glomerulus; AN, antennal nerve; cb, cell body cluster. Stars indicate AL hub. Directions: a, anterior; d, dorsal; l, lateral; m, medial; p, posterior; v, ventral. Scale bars = 50 μm.

The AL of *A*. *lucorum* is composed of a central fiber core, called AL hub, and a large number of roundish subcompartments, called antennal lobe glomeruli (ALG) (Fig 6). The glomeruli are arrayed roughly in a single layer around the hub. The diameter of the glomerular region is 139.38 ± 24.64 μm, 128.39 ± 8.7 μm, 95 ± 4.12 μm (mean ± SD, n = 5), in x, y, z direction, respectively. The volume of the AL is 274 342.25 ± 6517.34 μm^3^ (n = 5).

All the ALGs that could be identified were reconstructed. Generally, the glomeruli are relatively weakly separated, and for many glomeruli, the border is not obvious. Accurate identification of individual glomeruli across preparations is therefore difficult. The number of glomeruli was counted based on the three-dimensional reconstruction models. The numbers of glomeruli of one AL from four preparations were 71, 71, 78, and 80, respectively. The volumes of individual glomeruli vary in a large range from 499 μm^3^ to 9 092 μm^3^. The average volume of each glomerulus is 2756.16 μm^3^ (n = 71).

## Discussion

### Projection pathway of antennal sensory neurons in the central nervous system of *A*. *lucorum*

The CNS of *A*. *lucorum* is composed of highly fused ganglia, including the brain, GNG, proTG, and PG. In this study, we demonstrate further details including reconstruction of three dimensional neuropil structures of the CNS to provide a framework for visualization of the antennal sensory pathway.

Antennal sensory neurons of *A*. *lucorum* project into the brain via the ipsilateral antennal nerve and further down to the GNG, proTG, and PG. Such a projection pattern throughout the whole CNS was also observed in the blood-sucking bug *Rhodnius prolixus* [20]. The multiple targeting areas of the sensory axons indicate that the antenna of *A*. *lucorum* plays multiple roles, connected to olfaction and other modalities. This is in accordance with the types of antennal sensilla which were observed by using scanning electron microscopy [21]. On the antenna of *A*. *lucorum*, there are a large number of sensilla trichodea, sensilla basiconica, sensilla chaetica on flagellomeres, and some Böhm bristles on the pedicel and scape. It has previously been demonstrated that sensilla trichodea and sensilla basiconica house olfactory sensory neurons, sensilla chaetica contact chemosensory (gustatory) and mechanosensory neurons, and Böhm bristles mechanosensory neurons [22–26].

The antennal backfill staining was performed at the base of the scape. So all types of neurons located on the antenna could be stained. In the brain of *A*. *lucorum*, the antennal sensory neurons mainly project to the AL and the AMMC, which corresponds to the arrangement in other insects, for instance, mosquito [27], cockroach [28], psyllid [29], bugs [20, 29], honeybee [30], aphid [18], louse [31], cricket [32], dragonfly [33], moths [24, 26, 34]. The AL is the primary olfactory center of the insect and receives input from the olfactory antennal neurons [35]. The AMMC (also named the dorsal lobe in some insects, e.g. aphid, honeybee, cricket and cockroach) receives terminal arborizations from mechanosensory neurons located on the scape and pedicel [35]. So the projections in the AL of *A*. *lucorum* might originate from olfactory sensilla and those in the AMMC from mechanosensory sensilla. In the fruit fly and honeybee, the AMMC mainly receives mechanosensory neurons of Johnston’s organs serving as an auditory organ [36]. Also, in *Manduca sexta*, scolopidal neurons of Johnston’s organ located in the pedicel send their projections into the AMMC, here contributing to the control of flight stability [25].

The projections in the GNG of *A*. *lucorum* might originate from the sensory neurons of sensilla chaetica. Previous tip recording and individual tracing experiments have indicated that the sensilla chaetica of the moths *Heliothis virescens* and *Spodoptera littoralis* house three to four contact-chemosensory neurons, which project to the GNG [24, 26]. In addition, a sensillum chaeticum also houses one mechanosensory neuron in moths which projects to the AMMC. Projections in the tritocerebrum from this type of sensilla were also observed in the two moth species [24, 26]. However, no projections were observed in the tritocerebrum of *A*. *lucorum*.

The finding of a few sensory projections in the proTG and PG, as demonstrated here, has only been previously reported in the bug *R*. *prolixus* and the blowfly *Calliphora erythrocephala* [20, 37]. The pattern of terminal arborizations of *A*. *lucorum* is similar to that of *R*. *prolixus*, but somewhat different from that of *C*. *erythrocephala*. In *C*. *erythrocephala* these sensory neurons originate from the campaniform sensilla which are located on the pedicel [37]. In the thoracic ganglia their axons give off long bilateral branches into leg motor centers [37]. Such projection patterns of the antennal sensory neurons may suggest that the antenna plays an important role in controlling locomotion of the insect. As demonstrated here, the axons of antennal sensory neurons in the PG of *A*. *lucorum* reach as far as the AG. This is the first report about such innervations of antennal sensory neurons in the AG of insects. This kind of projection indicates that the antennal sensory neurons of *A*. *lucorum* may also play a certain role for the behavioral and physiological activities of the abdomen.

A few projections from antennal sensory neurons in *A*. *lucorum* were also observed in the ventral part of the posterior slope of the brain. A similar projection pattern has been found in the blowfly, fruit fly, and honeybee [36–38]. In these species, the sensory neurons, which target the ventral part of the posterior slope of the brain, originate from the mechanosensory sensilla which located on the pedicel, e.g. the campaniform sensilla in the blowfly and the Johnston’s organ in the fruit fly and honeybee [36–38]. It suggests that the ventral part of the posterior slope of the brain is also involved in mechanosensory perception.

### Glomerular organization of the antennal lobe of *A*. *lucorum*

As in many insect species, the AL of *A*. *lucorum* is composed of a large number of glomeruli. The number of glomeruli in one AL is mainly species-specific, for instance, about 50 glomeruli have been identified in dipteran species, 70 in lepidopteran, 160 in honeybee, 200–600 in ants, and more than 1000 mini glomeruli in locust [39, 40]. In some insect species, there are no glomeruli in the AL, for instance in Palaeoptera and anosmic insects, such as diving beetles and the back-swimmer [33, 39]. However, the number of glomeruli in hemipteran species varies considerably. There are about 70–80 glomeruli in *A*. *lucorum*, about 60–80 in the stink bug *Euschistus heros* [29], about 28 in *R*. *prolixus* [20], and 25–40 in the aphid *Acyrthosiphon pisum* [18]. In the psyllid, *Trioza apicalis*, and the aphids, *Sitobion avenae* and *Metopolophium dirhodum*, there are no evident glomeruli in the AL [29]. In contrast to anosmic insects, aphids and psyllids are very dependent on olfactory cues to find hosts. An aglomerular AL and ill-defined glomeruli in these insect species might result from low density of synapses or a small number of glia cells in the AL [29]. Glia cells play an important role in forming the glomeruli [41]. A vague AL structure may be a typical characteristic of Hemipteran insects [20].

In general, the shape, size, and location of each glomerulus in the AL of insects are unique [42]. Thus anatomical maps of the individual glomeruli have been made in many different insect species, particularly in moth species [34]. In this study, the presented three-dimensional AL model was created based on one single preparation mentioned in the result part. In *A*. *lucorum*, it is difficult to identify the same glomerulus across different individuals, due to the diffuse separation of the units. In future studies, it is necessary to stain the local interneurons and projection neurons of the AL. Such an approach has shown to be helpful for glomerular identification and for creating a complete glomerular map [34].

## Conclusions

Taken together, the backfilling staining on the antenna of *A*. *lucorum* reveals that the axons of the antennal sensory neurons project via the ipsilateral antennal nerve throughout the whole CNS, from the brain to the gnathal ganglion, prothoracic ganglion, mesothoracic ganglion, and metathoracic ganglion, and as far as to the abdominal ganglion. Such a projection pattern indicates that the antenna of *A*. *locurom* plays multiple roles in different areas of CNS, e.g. olfaction, gustation, mechanosensation, and others. The AL, however, is the major target area of antennal sensory neurons, which indicates that *A*. *locurom* is largely dependent on the olfaction. The number of glomeruli of *A*. *locurom* was roughly counted and the three dimensional of the AL was reconstructed. Such anatomic knowledge of the AL provides important information for further investigations of chemosensory encoding mechanisms of the mirid bug.
